# A checklist of the scorpions of Ecuador (Arachnida: Scorpiones), with notes on the distribution and medical significance of some species

**DOI:** 10.1186/s40409-015-0023-x

**Published:** 2015-07-30

**Authors:** Gabriel Brito, Adolfo Borges

**Affiliations:** Laboratory of Biotechnology, Faculty of Natural Sciences, University of Guayaquil, Guayaquil, Ecuador; Laboratorio de Biología Molecular de Toxinas y Receptores, Instituto de Medicina Experimental, Facultad de Medicina, Universidad Central de Venezuela, Caracas, Apartado Postal 50587, Venezuela; National Secretariat for Higher Education, Sciences, Technology and Innovation of Ecuador (Senescyt), Quito, Ecuador

**Keywords:** Scorpions, Ecuador, *Ananteris*, *Brachistosternus*, *Chactas*, *Centruroides*, *Hadruroides*, Scorpionism, *Teuthraustes*, *Tityus*, *Troglotayosicus*

## Abstract

Ecuador harbors one of the most diverse Neotropical scorpion faunas, hereby updated to 47 species contained within eight genera and five families, which inhabits the “Costa” (*n* = 17), “Sierra” (*n* = 34), “Oriente” (*n* = 16) and “Insular” (*n* = 2) biogeographical regions, corresponding to the western coastal, Andean, Amazonian, and the Galápagos archipelago regions, respectively. The genus *Tityus* Koch, in the family Buthidae, responsible for severe/fatal accidents elsewhere in northern South America and the Amazonia, is represented in Ecuador by 16 species, including *T. asthenes*, which has caused fatalities in Colombia and Panama, and now in the Ecuadorian provinces of Morona Santiago and Sucumbíos. Underestimation of the medical significance of scorpion envenoming in Ecuador arises from the fact that *Centruroides margaritatus* (Gervais) (family Buthidae) and *Teuthraustes atramentarius* Simon (family Chactidae), whose venoms show low toxicity towards vertebrates, frequently envenom humans in the highly populated Guayas and Pichincha provinces. This work also updates the local scorpion faunal endemicity (74.5 %) and its geographical distribution, and reviews available medical/biochemical information on each species in the light of the increasing problem of scorpionism in the country. A proposal is hereby put forward to classify the Ecuadorian scorpions based on their potential medical importance.

## Introduction

Ecuador, despite its small size (only 250,000 km^2^ or 1.5 % of South America), ranks 17^th^ among the nations of the world in biodiversity [1, 2]. This is attributable to, among other factors, the confluence of several biogeographic regions: Chocó and Tumbez (encompassing the “Costa” region), northern and south-central Andes (Sierra” region), the northern and southwestern Amazon (Oriente” region) and the insular region of Galápagos (Fig. 2). Ecuador harbors one of the richest arachnid faunas of the Neotropics [3]; specifically, its scorpion fauna ranks high amongst South American countries in terms of diversity, with 12.70 species per 100,000 km^2^ [4].

Pioneering work by Eugéne Simon, Reginald I. Pocock, Alfredo Borelli, Karl Kraepelin, and Cândido de Mello-Leitão initiated the cataloguing of Ecuadorian scorpions [5–13]. Although less known, the work of the Ecuadorian taxonomist Francisco Campos [14, 15], together with Behr-Castillo and Correa [16], also contributed to the knowledge of local scorpions, particularly in the province of Guayas. The work of Wilson R. Lourenço has allowed recognition of Ecuador as part of a region exhibiting the highest alpha-diversity for scorpions in the world, also encompassing Southern Colombia, the Northeast region of Peru, and the Upper Amazon region of Brazil [17].

Particularly, the genera *Tityus* C. L. Koch (in the family Buthidae) and *Teuthraustes* Simon (in the family Chactidae) show a high concentration of species in Ecuador [18, 19]. A center of endemism in Ecuador for *Teuthraustes* has been proposed based on its extreme local diversity [20]. Despite such diversity, little is known about the toxicity of local scorpions, particularly within the speciose genus *Tityus*, which contains all medically important species in South America, and exhibits a phylogenetic divergence paralleled by functional, structural, and immunological differences among their toxins [21, 22].

Mostly dating from records obtained at the turn of the 19^th^ century and from the 1900–1920 period, type localities for a number of Ecuadorian species are obscure. An example of this situation shown recently is the chactid *Chactas rubrolineatus* Simon, described by Eugène Simon from Rio Içá in 1880, which was wrongly assigned to Ecuador by Mello-Leitão [10] when in fact its location lies in Brazil [23].

The goal of this work is therefore to update the list of species and review the literature on scorpion Ecuadorian fauna from geographical and toxicological standpoints whenever the information is available. This idea arises, on one hand, from the increasing clinical relevance of some species in the country, with several fatal and severe infant cases reported from the provinces of Sucumbíos and Morona Santiago in 2012–2014 ([24]; Dr. Jorge Blanco, Fundación Herpetológica Gustavo Orcés, personal communication). Comprehension of the basis underlying the extreme diversity of the Ecuadorian scorpion fauna, particularly in the case of those species toxic to humans, necessarily relies on the correct assignment of collection localities.

**Fig. 1:**
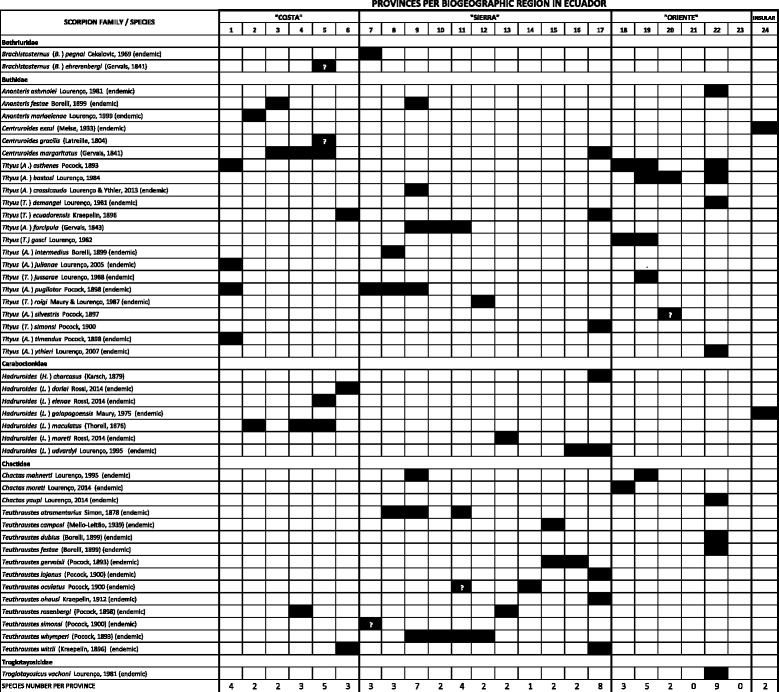
Distribution of scorpion species per political province and biogeographical area in Ecuador. Collection localities not yet defined are marked with question marks

**Fig. 2:**
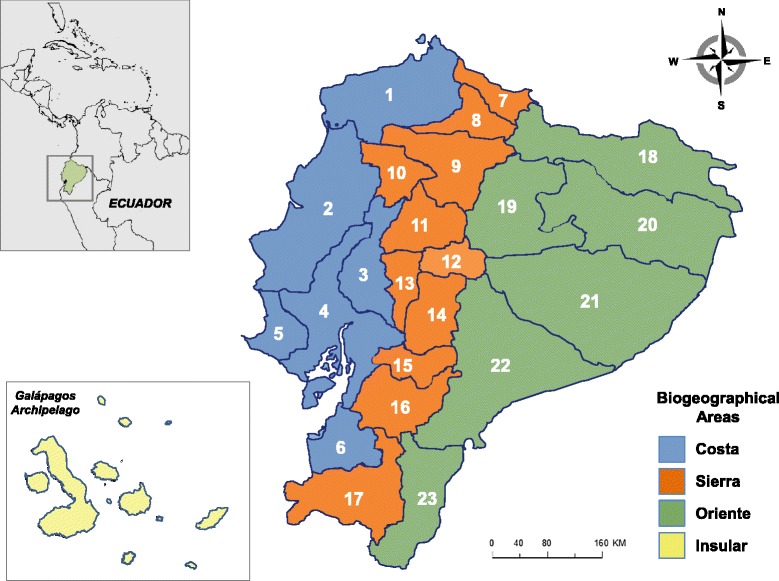
Political provinces of Ecuador and biogeographical regions encompassing such provinces. “Costa” region: (1) Esmeraldas, (2) Manabí, (3) Los Ríos, (4) Guayas, (5) Santa Elena, (6) El Oro. “Sierra” region: (7) Carchi, (8) Imbabura, (9) Pichincha, (10) Santo Domingo de los Tsáchilas, (11) Cotopaxi, (12) Tungurahua, (13) Bolívar, (14) Chimborazo, (15) Cañar, (16) Azuay, (17) Loja. “Oriente” región: (18) Sucumbíos, (19) Napo, (20) Francisco de Orellana, (21) Pastaza, (22) Morona Santiago, (23) Zamora Chinchipe. “Insular” region: Galálapagos islands

Here follows a list of the taxa currently recognized to inhabit Ecuador, including comments on their distribution and type localities, after confronting published collection sites with contemporary geographical names. We used various search engines for placing type localities (Google Earth, GeoNames). Taxa are organized in alphabetical order. Figure 1 summarizes the occurrence of individual species per province; the map in Fig. 2 identifies provinces in the contemporary political map of Ecuador. Information on the location of holotypes and syntypes for Ecuadorian species is available from the cited literature [17, 25–32]. We also review the potential medical importance of scorpion genera and species prevalent in Ecuador.

## Annotated list of Ecuadorian scorpion species

- Family Bothriuridae Simon, 1880
  - Genus *Brachistosternus* Pocock, 1893
    - *Brachistosternus* (*Brachistosternus*) *pegnai* Cekalovic, 1969
    - *Brachistosternus* (*Brachistosternus*) *ehrenbergii* (Gervais, 1841)

- Family Buthidae Simon, 1880
- Genus *Ananteris* Thorell, 1891
  - *Ananteris ashmolei* Lourenço, 1981
  - *Ananteris festae* Borelli, 1899
  - *Ananteris mariaelenae* Lourenço, 1999

- Genus *Centruroides* Marx, 1889
  - *Centruroides exsul* (Meise, 1933)
  - *Centruroides gracilis* (Latreille, 1804)
  - *Centruroides margaritatus* (Gervais, 1841)

- Genus *Tityus* C. L. Koch, 1836
  - *Tityus* (*Atreus*) *asthenes* Pocock, 1893
  - *Tityus* (*Archaeotityus*) *bastosi* Lourenço, 1984
  - *Tityus* (*Atreus*) *crassicauda* Lourenço & Ythier, 2013
  - *Tityus* (*Tityus*) *demangei* Lourenço, 1981
  - *Tityus* (*Tityus*) *ecuadorensis* Kraepelin, 1896
  - *Tityus* (*Atreus*) *forcipula* (Gervais, 1844)
  - *Tityus* (*Tityus*) *gasci* Lourenço, 1981
  - *Tityus* (*Archaeotityus*) *intermedius* Borelli, 1899
  - *Tityus* (*Archaeotityus*) *julianae* Lourenco, 2005
  - *Tityus* (*Tityus*) *jussarae* Lourenço, 1988
  - *Tityus* (*Atreus*) *pugilator* Pocock, 1898
  - *Tityus* (*Tityus*) *roigi* Maury & Lourenço, 1987
  - *Tityus* (*Tityus*) *simonsi* Pocock, 1900
  - *Tityus* (*Archaeotityus*) *silvestris* Pocock, 1897
  - *Tityus* (*Atreus*) *timendus* Pocock, 1898
  - *Tityus* (*Atreus*) *ythieri* Lourenço, 2007

- Family Caraboctonidae Kraepelin, 1905
- Genus *Hadruroides* Pocock, 1893
  - *Hadruroides (Hadruroides) charcasus* (Karsch, 1879)
  - *Hadruroides* (*Lourencoides*) *doriai* Rossi, 2014
  - *Hadruroides* (*Lourencoides*) *elenae* Rossi, 2014
  - *Hadruroides* (*Lourencoides*) *galapagoensis* Maury, 1974
  - *Hadruroides* (*Lourencoides*) *maculatus* (Thorell, 1876)
  - *Hadruroides* (*Lourencoides*) *moreti* Rossi, 2014
  - *Hadruroides* (*Lourencoides*) *udvardyi* Lourenço, 1995

- Family Chactidae Laurie, 1896
- Genus Chactas Gervais, 1844
  - *Chactas mahnerti* Lourenço, 1995
  - *Chactas moreti* Lourenço, 2014
  - *Chactas yaupi* Lourenço, 2014

- Genus *Teuthraustes* Simon, 1878
  - *Teuthraustes atramentarius* Simon, 1878
  - *Teuthraustes camposi* (Mello-Leitão, 1939)
  - *Teuthraustes dubius* (Borelli, 1899)
  - *Teuthraustes festae* (Borelli, 1899)
  - *Teuthraustes gervaisii* (Pocock, 1893)
  - *Teuthraustes lojanus* (Pocock, 1900)
  - *Teuthraustes oculatus* Pocock, 1900
  - *Teuthraustes ohausi* Kraepelin, 1912
  - *Teuthraustes rosenbergi* (Pocock, 1898)
  - *Teuthraustes simonsi* (Pocock, 1900)
  - *Teuthraustes whymperi* (Pocock, 1893)
  - *Teuthraustes wittii* (Kraepelin, 1896)

- Family Troglotayosicidae Lourenço, 1998
- Genus *Troglotayosicus* Lourenço, 1981
  - *Troglotayosicus vachoni* Lourenço, 1981

### Family Bothriuridae

#### Genus *Brachistosternus*

This genus is amply distributed in the coastal region of Peru and northern Chile, and comprises 41 species contained within two subgenera, *Brachistosternus* Pocock and *Ministernus* Francke [33]. Records for Ecuador include only two species, *Brachistosternus* (*Brachistosternus) pegnai* Cekalovic, from the province of Carchi, and *Brachistosternus* (*Brachistosternus) ehrenbergii* (Gervais), whose current distribution in the country is not known. Although the sting by some species, including *B.* (*B.*) *ehrenbergii*, can be painful to humans, there are no reports of fatal envenoming by taxa in this genus. In Peru, *B.* (*B.*) *ehrenbergii* is a highly abundant synanthropic scorpion along the coastline [34].

A 6.7 kDa toxin denominated Be1 has been isolated from the venom of *B.* (*B.*) *ehrenbergii* which produces salivary secretion and spastic paralysis in mice at a dose of 3 mg/kg upon intraperitoneal injection and is lethal after two hours [35]. Be1 only comprises 7 % (w/w) of the total venom protein content, in comparison with the typical 20-30 % content of low-molecular-weight neurotoxins in the venom from *Tityus* species [36], which probably explains the low lethal dose in mice of *B.* (*B.*) *ehrenbergii* crude venom (subcutaneous injection), which is around 20 mg/kg [34]. According to its molecular mass and physiological effects, Be1 may belong to the sodium channel-active family of scorpion toxins (NaScTx).

It is not known whether other *Brachistosternus* spp., including those prevalent in Ecuador, are of equally low toxicity to mice, but their medical importance requires evaluation considering the higher sensitivity of humans (five-fold more susceptible than mice) to scorpion venom [37]. As venom of another bothriurid from Australia, *Cercophonius squama* (Gervais), contains only primitive homologs of NaScTxs (lipolytic-like peptides), South American representatives of the family Bothriuridae (including those inhabiting Ecuador) might have diverged toxinologically from those genera prevalent in Southeast Asia [38].

1. *Brachistosternus* (*Brachistosternus) pegnai* Cekalovic, 1969:163–168.
  This species is reported in San Gabriel, “Carcha,” which actually corresponds to the province of Carchi, northern Ecuador [39]. In his original description, Cekalovic [25] does not specify the province of origin but provided a map that places the type locality roughly within Carchi. San Gabriel is at an altitude of 2,980 m (0.59318 N, 77.83078 W), well above the arid and semi-arid habitats of species within this genus prevalent in central Peru and northern Chile but related to Andean congenerics from Argentina, Chile, Bolivia, and Peru [40]. *B.* (*B.*) *pegnai* type locality is at the northernmost limit of the genus distribution range [33].

2. *Brachistosternus* (*Brachistosternus) ehrenbergii* (Gervais, 1841:285).
  *B.* (*B.*) *ehrenbergii* has been reported as present in Ecuador [41], although Ochoa and Ojanguren Affilastro [42] have verified its presence only from northern Chile to central Peru. Lourenço [18] places *B.* (*B.*) *ehrenbergii* Ecuadorian populations in the province of Santa Elena without providing precise locations and thus the species has been assigned to this province (Fig. 1), pending new findings in Ecuador.

### Family Buthidae

#### Genus *Ananteris*

This genus comprises one of the smallest Neotropical buthids, between 15 and 41 mm of total body length in the case of Ecuadorian species. They are clearly recognizable by their densely spotted pigmentation, elongated telson, and pectines without fulcra [41]. No venoms from this genus have ever been analyzed, although toxins from *Ananteris* spp. are predicted to be ancestral to toxins produced by *Tityus* spp. given the Gondwanian distribution of *Ananteris*, since extant species are found both in Africa and Central/South America [43].

1. *Ananteris ashmolei* Lourenço, 1981:644–648
  Collected from Cueva de los Tayos (“Cave of the Oilbirds”) (1.9333S, 77.7928 W), a natural cave located on the eastern slopes of the Andes, in the province of Morona Santiago [44].

2. *Ananteris festae* Borelli, 1899:1–4.
  Collected from Rio Peripa by Enrico Festa during his 1895–1898 trip to Ecuador [7]. Assigning a precise location to Festa’s site of collection is not possible since the provinces of Guayas, Manabí, Santo Domingo de los Tsáchilas, and Pichincha currently share this river. *A. festae* is reported from the provinces of Los Ríos (near Quevedo) and Pichincha, confirming its prevalence in the Inter-Andean valley region of Ecuador [19, 26, 45].

3. *Ananteris mariaelenae* Lourenço, 1999:97–99.
  Type material from 75–80 km northeast of Chone, northern section of the province of Manabí [26].

### Genus *Centruroides*

This genus contains three species in Ecuador. Venom from *Centruroides margaritatus* Gervais, the most common scorpion in the coastal region of Ecuador, is richer in toxins active against potassium channels and also antimicrobial peptides in comparison with the higher content of sodium channel-active toxins typically found in venoms of toxic *Centruroides* species inhabiting Mexico and the southern United States [46, 47], explaining the lower toxicity of *C. margaritatus* to vertebrates.

**Fig. 3:**
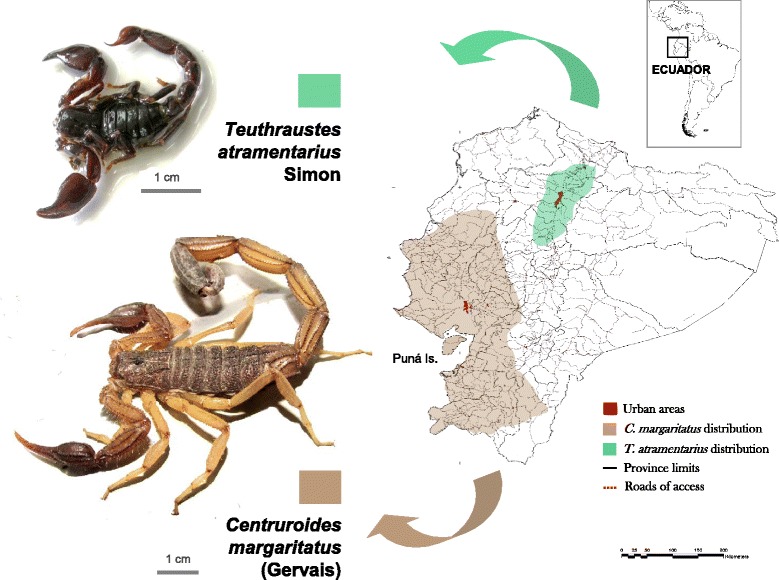
Distribution in Ecuador of synanthropic species *Teuthraustes atramentarius* Simon and *Centruroides margaritatus* (Gervais). Geographical ranges are taken from Fig. 1. *C. margaritatus* specimen is from Mapasingue County, Guayaquil (picture by Xavier Cornejo, Facultad de Ciencias Naturales, Universidad de Guayaquil). *T. atramentarius* specimen (collected in Quito, Pichincha Province) is from the collection of the Invertebrate Museum, Pontificia Universidad Católica del Ecuador (photograph by Tatiana Torres)

1. *Centruroides exsul* (Meise, 1933:27).
  This species is endemic to the Galápagos islands, the type material having been collected at Floreana Island (1.1751S, 90.263 W) [48]. Distribution: Islands of Española, Fernandina, Floreana, Marchena, Pinta, San Cristóbal, San Salvador, Santa Cruz, and Santiago. This species seems to be confined to the low dry arid zone, except for Pinta where it is found in evergreen forests [49].

2. *Centruroides gracilis* (Latreille, 1804:127).
  Lourenço [18] reports this species roughly within the province of Santa Elena but no specific locations are provided. Identity of true Ecuadorian *C. gracilis* populations is pending since records of this species in South America have been questioned [50].

3. *Centruroides margaritatus* (Gervais, 1841:281–282).
  This species (Fig. 3) is by far the most abundant scorpion along the Ecuadorian coast and responsible for the majority of envenoming cases in the metropolitan area of Guayaquil, province of Guayas, the most populated city of Ecuador [14,16,]. It is a large species (65–100 mm), with carapace and tergites dark yellow-brown and metasomal segments I–IV yellow-brown, darker on IV; V and telson dark reddish brown [50].

The type locality of this species is Isla Puná, at the entrance of the Gulf of Guayaquil, currently belonging to the province of Guayas [50]. This species has been reported from the coastal provinces of Los Ríos and Santa Elena, and the “Sierra” province of Loja (Fig. 1), but no data are available on its presence in the coastal provinces of Esmeraldas and El Oro [50]. The records for the “Sierra” provinces of Pichincha and Chimborazo date from 1901–1907 and have not been confirmed since [51]. This species has been the subject of controversy for more than a century in relation to its real range of distribution as has been frequently misidentified with its allied species, *Centruroides gracilis* (Latreille), and *Centruroides edwardsii* (Gervais). The matter was settled by Armas *et al*. [50] who differentiated *C. margaritatus* from its congeners based on its geographical distribution in South America and the Caribbean, and morphology (the lower hairiness of its pedipalps, which are more oval in shape, and the presence of only eight rows of denticles in the pedipalp fixed finger) [50].

The difficulties associated with the identification of true populations of *C. margaritatus* are probably the origin of the conflicting reports of its venom lethality towards vertebrates. Marinkelle and Stahnke [52] reported a median lethal dose (LD50) in mice of 59.9 mg/kg (intraperitoneally) from a *C. margaritatus* Colombian population, whereas Gómez *et al*. [53] has reported a lethal titer in mice of 5.19 mg/kg for the same species but from a Costa Rican population. Central American *C. margaritatus* populations now belong to *C. edwardsii* [50]. As to the Ecuadorian populations of *C. margaritatus*, venom from specimens collected in Guayaquil has been shown to decrease significantly the cardiac frequency when injected subcutaneously into rabbits [54]. Campos [14] also reports on severe manifestations on a human adult envenomed by *C. margaritatus* in Playas, province of Guayas, who experienced vomiting, intense dyspnea, shivering, and numbness of the tongue [14]. Despite these observations, no modern reports have been published on envenoming by *C. margaritatus* in Ecuador. One recent adult case of *C. margaritatus* envenoming in Guayaquil presented with paresthesia and erythema at the sting site (Dr. Miguel Delgado, Postgraduate Program in Intensive Care, Universidad Espíritu Santo, Guayaquil, personal communication).

#### Material collected

In peridomiciliary areas in the cities of Portoviejo (1.316S, 80.2716 W) (*n* = 7; 3♀♀, 4♂♂) and Chone (0.4153S, 80.537 W) (*n* = 10; 6♀♀, 4♂♂), both in the lowland area of the province of Manabí (14.XII.2014, A. Borges coll). These findings extend *C. margaritatus* distribution range to this province; mild envenomings by this species are common in both cities. Specimens of *C. margaritatus* have also been collected in the metropolitan areas of Quevedo (1.02863S, 79.46352 W) (*n* = 10, 2♀♀, 8♂♂; 5.VIII.2014, T. Escobar coll.), and Babahoyo (1.80217S, 79.53443 W) (*n* = 10, 3♀, 7♂; 20.VIII.2014, T. Escobar coll.), both in the province of Los Ríos.

### Genus *Tityus*

*Tityus* is unquestionably the most complex genus of scorpions from a taxonomical standpoint (over 200 described species), accountable for the majority of severe and lethal scorpionism cases in Central and South America and the Caribbean, including Ecuador, due to the high lethality of their venoms towards vertebrates (intraperitoneal LD50 for mice ranging from 0.7 to 12 mg/kg) [22, 47, 55–57]. The genus has been divided into the subgenera *Archaeotityus* Lourenço*, Atreus* Gervais, *Brazilotityus* Lourenço, and *Tityus* Koch to accommodate taxa previously assigned to the morphological groups “*androcottoides*,” “*asthenes*,” “*bahiensis*,” “*bolivianus*,” “*clathratus*,” “*crassimanus,” “forcipula,” “melanostictus”* and *“quisqueyanus”* [58].

**Fig. 4:**
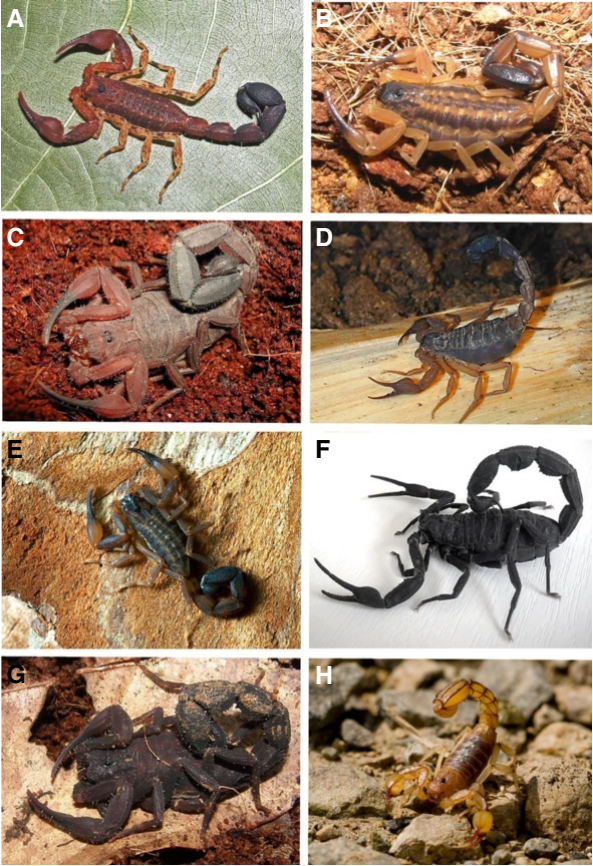
*Tityus* and *Hadruroides* species inhabiting Ecuador. **a** *Tityus* (*A.*) *bastosi* – subgenus *Archaeotityus* (male, picture taken by Richard C. West); **b** *Tityus* (*T.*) *ecuadorensis* - “*Tityus bolivianus*” group (female, picture taken by Michiel Cozijn); **c** *Tityus* (*A.*) *ythieri* – “*Tityus androcottoides*” group (male, picture taken by Eric Ythier); **d** *Tityus* (*T.*) *pugilator* – “*Tityus bolivianus*” group (male, picture by Jan Ove Rein); **e** *Tityus* (*T*.) *roigi* – “*Tityus bolivianus*” group (male from Baños de Agua Santa, Tungurahua province, picture by Radomir Jirsak), **f** *Tityus* (*A.*) *asthenes* – “*Tityus asthenes*” group (male from Shushufindi, province of Sucumbíos, picture by A. Borges; **g** *Tityus* (*A.*) *forcipula* – “*Tityus forcipula*” group (female, picture taken by Michiel Cozijn); **h** *Hadruroides* (*L*.) *charcasus* (Karsch) (from Macará, El Oro province, Ecuador; picture by Radomir Jirsak)

In Ecuador, the majority of *Tityus* species (*n* = 16) belong to subgenera *Atreus* (*n* = 5) and *Tityus* (*n* = 7), with four taxa corresponding to the subgenus *Archaeotityus*. Species within *Atreus* belong to either the morphological groups “*Tityus forcipula*” (*n* = 2), “*Tityus asthenes*” (*n* = 2), or “*Tityus androcottoides*” (*n* = 1) whereas all species within the subgenus *Tityus* belong to the “*Tityus bolivianus*” complex. Described Ecuadorian *Tityus* spp. are mostly from the “Sierra” (*n* = 9) and “Oriente” provinces (*n* = 7), with only one record – *Tityus* (*Atreus*) *asthenes* Pocock – from the coastal province of Esmeraldas (Fig. 1). Some representatives of the Ecuadorian *Tityus* fauna are depicted in Fig. 4. We have excluded *Tityus* (*Atreus*) *spinatus* Pocock from the list of Ecuadorian species since this was synonymized with *Tityus* (*Atreus*) *forcipula* Pocock by Lourenço [59] and was not included by Fet *et al.* [41] in their catalog of world scorpions. Lourenço and Ythier [17] indicated, however, that *T.* (*A.*) *spinatus* is a species associated with the “*Tityus forcipula*” group but provided no details regarding its validity [17].

1. *Tityus* (*Atreus*) *asthenes* Pocock, 1893:313.
  This species (Fig. 4 – f) was described from a specimen collected in “Pororu,” Peru, by Pocock [60], but no contemporary locality with this name exists in this country. It has been suggested that the correct locality is Paruro, in the department of Puno, southern Peru [61], although no modern records of *T.* (*A.*) *asthenes* exist for this region, where *Tityus* (*Tityus*) *soratensis* Kraepelin is the only *Tityus* species reported thus far [62]. More recent Peruvian records for *T.* (*A.*) *asthenes* are supplied by Teruel [63], from Capahuari, province of Loreto, on the border with Ecuador. *T.* (*A.*) *asthenes* presents a disjointed distribution in Ecuador (*cis*- and *trans*-Andean), since it inhabits the western and eastern versants of the Andes range, having been reported from the western province of Esmeraldas and the eastern province of Napo [18].
  Considering its abundance in the areas where accidents have taken place, *T*. (*A*.) *asthenes* is probably the species associated with the severe and fatal accidents in children from the provinces of Sucumbíos and Morona Santiago (A.B., unpublished observations). *T.* (*A.*) *asthenes* has been found from Peru to Panama, having produced severe envenoming cases and fatalities in Colombia [64] and Panama [47, 65]. *T.* (*A.*) *asthenes* neurotoxins from the Panamanian populations are only weakly recognized by the anti-*Tityus (Tityus) serrulatus* (Brazil) and anti-*Tityus* (*Atreus*) *discrepans* (Venezuela) antivenoms [66]. A test of the immunological reactivity of venom from Ecuadorian populations of *T.* (*A.*) *asthenes* towards available antivenoms is pending.

#### Material collected

Shushufindi, province of Sucumbíos (*n* = 5, 2♀♀, 3♂♂; 31/VII/2014, inside rural housing, T. Escobar coll.), thereby extending *T. asthenes* distribution range to this province in Ecuador.

2. *Tityus* (*Archaeotityus*) *bastosi* Lourenço, 1984:358–359.
  This species (Fig. 4 - a) belongs to the subgenus *Archaeotityus*, which comprises highly pigmented scorpions (formerly in the “*Tityus clathratus*” morphological group), the smallest (18–40 mm) and most ancient group of species in this genus, in which the subaculear tooth is always rhomboidal. Type material of *T. bastosi* is from Los Tayos, province of Morona Santiago; also reported from Coca (Puerto Francisco de Orellana, 0.46289S, 76.9868 W), province of Napo, amply distributed in the Amazonian regions of Brazil (São Paulo de Olivença, state of Amazonas) and Peru (Iquitos, department of Loreto) [67].

3. *Tityus* (*Atreus*) *crassicauda* Lourenço & Ythier, 2013:2–9.
  Type material from Tandayapa (0.01667S, 78.76667 W), Pichincha Province [17]. *T. crassicauda* belongs to the “*Tityus forcípula*” morphological group (in the subgenus *Atreus*), which comprises medium- to large-sized (above 50 mm), reddish-brown (in adults) species, characterized by the presence of strong spinoid granules at the end of dorsal metasomal carinae [59]. The group encompasses several *Tityus* spp. such as *Tityus* (*Atreus*) *fuhrmanni* Kraepelin, *Tityus* (*Atreus*) *metuendus* Pocock, *Tityus* (*Atreus*) *pachyurus* Pocock, *Tityus* (*Atreus*) *macrochirus* Pocock, and *Tityus* (*Atreus*) *festae* Borelli, all [with the exception of *T.* (*A.*) *macrochirus*] known to be responsible for high morbidity and pediatric mortality across their distribution range in Panama, Colombia, and Peru [22, 47, 56]. The epidemiological status of the Ecuadorian species in the “*Tityus forcipula*” group has not yet been determined.

4. *Tityus* (*Tityus*) *demangei* Lourenço, 1981:640–644.
  Holotype from Cueva de los Tayos, province of Morona Santiago. This species belongs to the “*Tityus bolivianus*” morphological group (in the subgenus *Tityus*), which includes medium to large-sized (40–70 mm), yellowish/chestnut brown species, distributed along the foothills of the Andes, from Ecuador to northern Argentina and Uruguay [68].

5. *Tityus* (*Tityus*) *ecuadorensis* Kraepelin, 1896:127–129.
  Fet *et al*. [41] reported Piscobamba, in the province of Loja, Ecuador, as the type locality for this species. There is no such locality in Ecuador but rather in the Peruvian province of Áncash. Instead, the river Piscobamba (4.1447S, 79.1756 W), may be the site of type collection in Ecuador, located east of Malacatos, southern section of the province of Loja. Also reported from Landangui (4.20521S, 79.22455 W) (Loja province) and Zaruma (El Oro province) (3.69132S, 79.61174 W). This species (Fig. 4 ‐ b) also belongs to the “*Tityus bolivianus*” complex and is a shared species with Peru, where has been collected in the departments of Cajamarca and Cuzco [68].

6. *Tityus* (*Atreus*) *forcipula* (Gervais, 1844:130)
  Type material from an unknown locality in Colombia [41]. This species (Fig. 4) is distributed from southwestern Colombia (department of Cauca) to northwestern Ecuador (provinces of Pichincha, Santo Domingo de Tsáchilas, and Cotopaxi) [18, 69]. *T.* (*A.*) *forcipula* is the type species of the “*Tityus forcipula*” morphological group.

7. *Tityus* (*Tityus*) *gasci* Lourenço, 1981:841–845.
  Holotype from southern French Guyana [70]. The species is reported from Cuyabeno, in the province of Napo, a locality that currently lies in the province of Sucumbíos (0.25911S, 75.89398 W). The species is amply distributed in the Amazon Basin, from French Guyana and Brazil to Ecuador and Peru.

8. *Tityus* (*Archaeotityus*) *intermedius* Borelli, 1899:8–10.
  Type material from the city of Ibarra, province of Imbabura, northern Ecuador. This species has been reported from Colombia but it has not been included on recent lists of Colombian *Tityus* species and is hereby considered endemic in Ecuador [10, 69, 71].

9. *Tityus* (*Archaeotityus*) *julianae* Lourenço, 2005:222–224.
  Holotype from the Valley of river Cayapas, west of San Miguel, province of Esmeraldas (0.7436S, 78.91506 W) [27].

10. * Tityus* (*Tityus*) *jussarae* Lourenço, 1988:686–688.
  Holotype from Cueva del Lagarto, near Mondayacu (0.4936S, 77.4617 W), province of Napo, and also from Cueva San Bernardo, in the same province. The species belongs to the “*Tityus bolivianus*” group of species.

11. * Tityus* (*Tityus*) *pugilator* Pocock, 1898:413–414.
  Holotype from “Cachavi,” Ecuador. The correct locality is actually San Javier de Cachaví, northwestern section of the Esmeraldas province (1.0358 N, 78.4638 W). Also collected from near the cave of Rumichaca de la Paz (“La Gruta de la Paz,” 0.301797 N, 77.50481 W), 18 km north of San Gabriel, in the province of Carchi, Cumbayá (valley of Tumbaco) (province of Pichincha), river Guayllabamba, near Quito (province of Pichincha), and from Ibarra (province of Imbabura) [18, 19] (Fig. 4). It belongs to the “*Tityus bolivianus*” species complex.

12. * Tityus* (*Tityus*) *roigi* Maury & Lourenço, 1987:80–84.
  Holotype collected in Baños (currently Baños de Agua Santa, 1.39639S, 78.42472 W), province of Tungurahua [72, 73]. The species belongs to the “*Tityus bolivianus*” complex.

13. * Tityus* (*Archaeotityus*) *silvestris* Pocock, 1897:363–364.
  The original description of this species was based on a specimen collected in Santarém, state of Pará, Brazil [74]. It is distributed along the Amazon basin from French Guyana, and has been reported in Ecuador although no precise collection localities have been mentioned [17, 75]. In the maps published by Lourenço *et al.* [17, 27], *T. silvestris* is roughly assigned to the eastern section of the province of Orellana.

14. * Tityus* (*Tityus*) *simonsi* Pocock, 1900:469–470.
  Type material is from the city of Loja, province of Loja [6]; the species has also been reported in Vilcabamba, province of Loja (4.26233S, 79.22287 W) [76]. This species is shared with northern Peru [68]. It is a very close taxon to *T.* (*T.*) *ecuadorensis* to the point that hybridization experiments have been suggested to determine whether *T. simonsi* could be a morph of the latter species [68].

15. *Tityus* (*Atreus*) *timendus* Pocock, 1898:414.
  Type material from San Javier de Cachaví, Esmeraldas province. Although restored by Lourenço [77], *T.* (*A.*) *timendus* was not included in a recent revision of the Ecuadorian *Tityus* fauna [17]. Thus, the species is included on the list of current species from Ecuador (Fig. 2). *T.* (*A.*) *timendus* has been placed in the “*Tityus asthenes*” species complex in the subgeneus *Atreus*, but has also been cited as an allied species of *T.* (*A.*) *pachyurus* [77], which is responsible for severe envenomings in Colombia and Panama [47, 78].

16. *Tityus* (*Atreus*) *ythieri* Lourenço, 2007:377–380.
  Holotype from south of Yaupi (2.85444S, 77.936 W), province of Morona Santiago (Fig. 4). It is the only *Tityus* species thus far described from Ecuador that belongs to the “*Tityus androcottoides*” morphological group, which comprises scorpions with ventral keels of metasomal segments II to IV partly or largely fused [28]. Species within this group are generally restricted to the east of the Colombian Eastern Cordillera and mainly inhabit the Venezuelan mountainous range [79] and a few have been described from the Brazilian Amazonia [*Tityus* (*Atreus*) *elizabethae* Lourenço & Ramos, *Tityus* (*Atreus*) *neblina* Lourenço] and eastern Colombia [*Tityus* (*Atreus*) *rebierei* Lourenço] [28, 71].

Most species within this group produce highly toxic venoms and are accountable for severe/lethal scorpionism, mainly in Venezuela [22]. Kovařík *et al.* [76] synonymized *T.* (*A.*) *ythieri* to *Tityus* (*Atreus*) *magnimanus* Pocock, based on a genetic and morphological comparison, but the species was later reinstated by Ythier [80] on the basis of a misidentification of the *T.* (*A.*) *ythieri* specimens studied by Kovařík *et al.* [76].

### Family Caraboctonidae

#### Genus *Hadruroides*

Species of *Hadruroides* (*n* = 22) are restricted to Ecuador, Peru, northern Chile, and several offshore islands (including the Galápagos), where they inhabit inter-Andean valleys, Pacific deserts, and dry forest habitats [32, 81]. In Ecuador the genus is represented by seven species, recently split into two subgenera by Rossi [32]. The nominal subgenus, *Hadruroides*, now includes only one species, *Hadruroides (Hadruroides) charcasus* (Karsch), whereas the subgenus *Lourencoides* Rossi includes all other known species. The total number of *Hadruroides* species inhabiting mainland Ecuador is now raised to six [32].

Ecuadorians records of *Hadruroides* (*Lourencoides*) *lunatus* (Koch) [82] are suggested to be probable misidentifications of other *Hadruroides* species, which is also the case in reports of this species from northern Chile [81]. Records of *Hadruroides* (*Lourencoides*) *leopardus* Pocock from the city of Loja, province of Loja [18], are probably referencing *Hadruroides* (*Lourencoides*) *udvardyi* Lourenço [81].

Species in this genus appear to be weakly toxic to vertebrates upon peripheral venom injection, as shown in the case of *H.* (*L.*) *lunatus* (from Lima, Peru) based on the low lethality (LD50 = 22 mg/kg) of its venom when injected intraperitoneally in mice. *H.* (*L.*) *lunatus* venom is, however, highly toxic upon central injection (LD50 = 0.1 mg/kg) [83]. Although *Hadruroides* venoms are not lethal to mammals, at least peripherally, they probably contain components able to elicit heart muscle disruption in vertebrates.

Serum levels of total creatine kinase (CK) and its isoenzyme MB (CK–MB), two widely accepted markers for heart injury or myocardial infarction, have been found significantly elevated in rats envenomed intraperitoneally with a sublethal dose of *H.* (*L.*) *lunatus* venom (5.8 mg/kg) [83]. Venom from another species, *H.* (*H.*) *charcasus* (Karsch) (from northern Peru), produces in the heart of *Bufo spinulosus* (Amphibia, Bufonidae) cellular necrosis, leukocyte infiltration, and endothelial lesions without significantly altering the cardiac force or frequency [84]. The actual clinical implications of these results are not yet clear, but these activities are probably due to the presence in venoms from *Hadruroides* spp. of low-molecular-weight neurotoxins and/or phospholipase components [83].

1. *Hadruroides* (*Hadruorides*) *charcasus* (Karsch, 1879:135)
  Holotype from Bolivia, from an unknown locality [41]. The species was redescribed by Maury [82] using material collected in the province of Cajamarca, northern Peru [82]. Rossi [32] reports this species from Macará, province of Loja (4.38181S, 79.9437 W) (Fig. 4).

2. *Hadruroides* (*Lourencoides*) *doriai* Rossi, 2014:206–207.
  Type material from San Pedro, county of Arenillas, El Oro province, in the border with Peru (3.66671S, 80.10142 W).

3. *Hadruroides* (*Lourencoides*) *elenae* Rossi, 2014:198–202.
  Holotype from La Puntilla, near Salinas (2.18973S, 81.01074 W), province of Santa Elena.

4. *Hadruroides* (*Lourencoides*) *galapagoensis* Maury, 1974:19.
  Endemic to the Galápagos islands [81]. Reported from the islands of Española, Fernandina, Floreana, Isabela, Pinzón, Santa Cruz, Santa Fé, Santiago, and San Salvador [85, 86]. *H*. (*L*.) *galapagoensis* is found from the coastal arid zone up to the arid top zone of the Isabela volcanoes [49].

5. *Hadruroides* (*Lourencoides*) *maculatus* (Thorell, 1876:186).
  Distributed in Ecuador on its central coastline, provinces of Manabí and Guayas, and shared with Peru (holotype from El Callao), *H*. (*L*.) *maculatus* seems to be the most common species of the genus in Ecuador [32, 81, 87]. It has been reported from Manta, and “Machalillo” [correct location is Machalilla (1.2824S, 80.4551 W)], both in the province of Manabí; La Puntilla, province of Santa Elena, and also from the city of Guayaquil, in the Estero del Salado (2.2533S, 80.210 W) [82]. Playas del Morro was mentioned by Campos [14] as a locality where he collected this species in the province of Guayas.

6. *Hadruroides* (*Lourencoides*) *moreti* Rossi, 2014:202–205.
  Type material from San Vicente, province of Bolìvar (1.90818S, 79.24859 W).

7. *Hadruroides* (*Lourencoides*) *udvardyi* Lourenço, 1995:76–78.
  Holotype from the province of Azuay, 90 km in the road from Cuenca to Loja. Endemic to inter-Andean valleys at altitudes above 2,000 m in southern Ecuador, in the provinces of Azuay and Loja [32, 81].

### Family Chactidae

#### Genus *Chactas*

This exclusively Neotropical genus is distributed from Costa Rica to northern Peru, with Colombia as a possible center of dispersion since most known *Chactas* species are from this country. A number of species have been described in Venezuela and isolated taxa are reported in Brazil and Peru. Species are also known from Costa Rica, Panama and the Island of Trinidad [29]. There are no species within this genus analyzed from either toxinological or clinical standpoints. The venom from the Neotropical chactid *Brotheas amazonicus* Lourenço possesses an LD50 in mice (intraperitoneally) of 90 mg/kg, two orders of magnitude above the lethality of venoms from *Tityus* spp. and twice the lethality of venoms from species in genera *Brachistosternus* and *Hadruroides* [57].

1. *Chactas mahnerti* Lourenço, 1995:70–71.
  Known from La Florida (0.220S, 78.300 W) and San Antonio (0.023S, 78.2648 W), province of Pichincha, and also from Coca, province of Napo [18].

2. *Chactas moreti* Lourenço, 2014:19–23.
  Type material collected at San Pablo de Kantesiya (0.1515S, 76.2535 W), near the Aguarico River, province of “Sucumbíus” [sic] (Sucumbíos, northern Ecuador) [29].

3. *Chactas yaupi* Lourenço, 2014:173–177.
  Collected north of the town of Yaupi (2.5116S, 77.569 W), between Yaupi and Morona, province of Morona Santiago [31].

### Genus *Teuthraustes*

All the species of *Teuthraustes* so far described (*n* = 24) have been collected in the Andean mountains of Ecuador, Peru and Colombia, and in the Amazonian highlands of Venezuela and Brazil, with the highest species diversity corresponding to Ecuador. Even though the taxonomic validity of some of these taxa will probably be the subject of further scrutiny, the outstanding concentration of species in Ecuador is realistic [20]. In their transferring of *Chactas camposi* Mello-Leitão to the genus *Teuthraustes*, Ochoa and Pinto da Rocha [30] presented a list of the 11 Ecuadorian species thus far reported, together with their provinces of origin. The following account clarifies the origin of some of these species and their geographical distribution.

1. *Teuthraustes atramentarius* Simon, 1878:400.
  This species is widespread in the provinces of Cotopaxi, Ibarra, and Pichincha (Fig. 3), and is certainly the most abundant scorpion in the metropolitan area of Quito [88]. Together with *C. margaritatus* from the coastal region, the abundance of these two species in the most industrialized and populated areas of Ecuador (i.e. Pichincha and Guayas provinces) suggests their responsibility for most scorpion envenomings in these regions. The fact that their venoms are not significantly toxic to vertebrates [52, 89] has been taken to indicate that scorpions are not of medical significance in the country, a situation reminiscent of the weakly toxic, widespread Venezuelan species, *Rhopalurus laticauda* (Thorell) [79]. In fact, *T. atramentarius* envenoming in humans only produces local symptomatology, resembling a bee sting [90].

2. *Teuthraustes camposi* (Mello-Leitão, 1939:147–148).
  The type locality for this species has been assigned by Ochoa and Pinto da Rocha [30] to the province of Cañar, northern Ecuador, but the precise location is not known. The type material of *T. camposi* was donated by the Ecuadorian entomologist Francisco Campos to C. de Mello-Leitão, at the Rio de Janeiro National Museum (MNRJ), who identified it as belonging to the genus *Chactas* [10]. Ochoa and Pinto da Rocha [30] later assigned the specimen to the genus *Teuthraustes* and stated that the type material at the MNRJ was originally labeled “*Chactas rosenbergi* Pocock.” In his 1931 account of Ecuadorian scorpions, Campos reports that the material classified as *Chactas rosenbergi* Pocock was collected in Bucay and Chimbo, in the provinces of Guayas and Bolívar, respectively, and that samples of “*Chactas* sp.” were obtained at Gualea (0.11677 N, 78.7476 W), in the province of Pichincha. There is no mention in his review of collection sites in the province of Cañar [14]. A further search for this species in the provinces of Cañar, Bolívar, Guayas, and Pichincha is pending to determine its true distribution range.

3. *Teuthraustes dubius* (Borelli, 1899:14).
  The type locality (“Valle de Santiago”) for this species was placed by Borelli [7] in “eastern Ecuador” without further details on its location. Borelli´s locality most probably refers to the channel of river Santiago, which originates in Ecuador from the union of two rivers, the Namangoza and the Upano, in the province of Morona Santiago, Ecuadorian Amazonia, where it flows 55 km before reaching the current border with Peru. The collector, Italian zoologist Enrico Festa, commented that the places where he collected in the Valle del Santiago were not far from the Marañón River, now belonging to Peru [90]. Back in the 1890s the area between the two rivers was under the jurisdiction of Ecuador. The species range of distribution includes the Cueva de los Tayos, also in Morona Santiago [44].

4. *Teuthraustes festae* (Borelli, 1899:11).
  This species was also collected by Enrico Festa in Valle del Santiago, province of Morona Santiago.

5. *Teuthraustes gervaisii* (Pocock, 1893:82).
  Type material from the city of Cuenca, the capital of the Azuay province [41, 60]. Also collected in the ruins of Ingapirca (2.54435S, 78.87741 W), between Cañar and El Tambo, province of Cañar, at 2,800 m altitude [91].

6. *Teuthraustes lojanus* (Pocock, 1900:472–473).
  Holotype collected from Loja, capital city of the province of Loja.

7. *Teuthraustes oculatus* Pocock, 1900:473.
  Type material collected from Sinche and Riobamba [6, 41]. Several towns named “Insinche” in Ecuador from the province of Cotopaxi, north of the province of Chimborazo (where the second locality, Riobamba, is placed), are candidates for the first locality. To the best of our knowledge, no records of this species exist from the Tungurahua province, as reported by Ochoa and Pinto da Rocha [30].

8. *Teuthraustes ohausi* Kraepelin, 1912:73,77-78.
  In his description of the species, Kraepelin [8], and also Fet *et al*. [41], refer to “Calamayo,” as its type locality, but the correct location is Catamayo (3.5911S, 79.2132 W), 36 km west of Loja, province of Loja.

9. *Teuthraustes rosenbergi* (Pocock, 1898:419–420).
  Holotype from Chimbo, near Guayaquil. Pocock [5] refers “Chimbo” as the type locality for this species, which actually corresponds to San José de Chimbo (1.410S, 79.133 W), province of Bolívar, 16 km south of Guaranda, the provincial capital. Campos [14] also reports this species from Bucay (2.100S, 79.600 W), province of Guayas, 100 km west of Guayaquil. He reports that *T. rosenbergi* is very abundant under the bark of fallen logs, together with insects from the order Dermaptera.

10. *Teuthraustes simonsi* (Pocock, 1900:471–472).
  Holotype from “Río Amboque,” Ecuador. This locality, given by Pocock [6] without further details, has no contemporary counterpart in Ecuadorian geography. Ochoa and Pinto da Rocha [30] assigned this species to the province of Carchi but an exhaustive search of current towns and rivers in this province has not rendered any similarities to this name.

11. *Teuthraustes whymperi* (Pocock, 1893:90).
  The type locality, “Millegalli,” is in fact the town of Nanegalito (0.356 N, 78.4048 W), in the northwestern section of the province of Pichincha. According to Lourenço [18], *T. whymperi* is a highly abundant scorpion in Las Pampas, northern section of the province of Cotopaxi. Also reported from the province of Pichincha [18, 30] and from Santo Domingo de los Tsáchilas and Tungurahua provinces [18].

12. *Teuthraustes wittii* (Kraepelin, 1896:141–144).
  As in the case of *Tityus* (*T*.) *ecuadorensis*, “Piscobamba” is referenced as the type locality for this species, but instead the river Piscobamba is the probable site of collection in the province of Loja. This species has also been reported from the cities of Loja and Vilcabamba, also in the Loja province, and from Zaruma (3.4128S, 79.3642 W), province of El Oro [18].

### Family Troglotayosicidae

#### Genus *Troglotayosicus*

The genus includes two troglomorphic species (i.e., with morphological adaptations to life in cavernicolous habitats, e.g., the absence of median eyes), one described from Ecuador and a second from the neighboring Colombian department of Nariño, *Troglotayosicus humiculum* Botero-Trujillo & Francke, 2009 [92]. Medical significance of species in this genus is probably poor given their restricted cavernicolous distribution.

1. *Troglotayosicus vachoni* Lourenço, 1981:651–656.
  Holotype found in Cueva de los Tayos, province of Morona Santiago [44].

## Concluding remarks

Surrounded by areas where scorpion stings are frequent and usually inflicted by noxious species, such as southern Colombia and the Brazilian Amazonia, there was a need to update the list of the scorpion fauna from Ecuador, and establish their geographic distribution and potential medical significance in light of recent accidents in children, some of them fatal [93, 56]. As noted before, insufficient efforts have been made, particularly in the case of the genus *Tityus*, to correlate human incidents and the precise geographical distribution of the species involved [94]. Preparation of a risk map for scorpionism in Ecuador and the possible manufacturing of a scorpion antivenom effective in the country would be facilitated by establishing such a correlation.

This work updates the number of Ecuadorian scorpion species to 47 and clarifies their distribution by biogeographic area and political provinces upon a thorough revision of individual collection localities based on contemporary records, summarized in Fig. 1. Such clarification should be helpful in future re-collections of specimens. This work also raises the number of endemic taxa to 35 (74.5 % of endemism), a rate comparable to that of Colombia (75.6 %) and only surpassed in northern South America by Venezuela (91.3 %) [79, 95]. For instance, Ecuador has been recognized as the probable center of dispersion for the scorpion genus *Teuthraustes*, in the family Chactidae, with half of the described species (*n* = 12) being endemic to the country [20]. Such dynamic speciation of Ecuadorian scorpions has been attributed to an evolutionary mode involving genetic drift in small founder populations, as the plant genera *Gasteranthus* (Gesneriaceae) and *Anthurium* (Araceae), with 25 and 50 %, respectively, of their world total species endemic to the environs of Ecuador [17, 96–98].

The incidence of scorpion stings in endemic areas is the result, among other factors, of the distribution areas of noxious species, their local abundance and ecology. The most speciose scorpion genera in Ecuador are *Tityus* (16 spp.), *Teuthraustes* (12 spp.), and *Hadruroides* (7 spp.), followed by *Ananteris* (3 spp.), *Brachistosternus* (2 spp.) and *Centruroides* (2 spp.), notwithstanding differences in their relative abundance among the biogeographical areas of “Costa,” “Sierra” and “Oriente” (Fig. 2). Scorpions in the genus *Hadruroides* are very abundant along the hyperxerophitic coastal areas (in provinces of El Oro, Guayas, and Santa Elena) and can be found under stones and dry manure [14]. There are no reports on the abundance of Ecuadorian *Brachistosternus* spp. but they are common in dunes of the Peruvian central and northern coast and should present a similar ecology in Ecuador, at least in the case of *B*. (*B*.) *ehrenbergii* [34]. Species in the genus *Teuthraustes* in Ecuador are mostly forest-dwelling taxa, native to the inter-Andean valleys, and are not hazardous to humans as shown in the case of *T. atramentarius*, a synanthropic, abundant species in the area of Quito, province of Pichincha [88, 89]. As stated above, *C. margaritatus* is the most common species found in domiciliary environments along the Ecuadorian coast and in populated areas such as Guayaquil and Milagro (province of Guayas), Babahoyo and Quevedo (province of Los Ríos), Portoviejo, Manta, and Chone (province of Manabí). *Ananteris* spp. are mostly sylvatic species. Specimens of *Tityus* spp. are abundant in domiciliary and peridomiciliary habitats of rural communities located in tropical and subtropical rain forest areas of Sucumbíos and Morona Santiago (“Oriente” area) and also in the province of Esmeraldas (“Costa” area), where they have been responsible for severe and lethal cases of scorpionism.

Traditionally, scorpions were not considered dangerous in Ecuador and their sting was supposedly fiercer if specimens from the coastal areas were involved, based on the notion that species from drier places produce venoms with higher toxicity [99]. Regardless of the species, envenoming manifestations in humans, which were claimed to be predominantly local, were reported to resemble those derived from wasp or bee stings [99]. Such an assumption, which has prevailed in modern times, is a consequence of the low toxicity towards vertebrates of the venoms produced by *C. margaritatus* and *T. atramentarius*, which are the most common urban species in the country. It is clear from the above account that noxious scorpion species inhabit Ecuador and are capable of producing significant morbidity and pediatric mortality.

According to the species involved, their areas of distribution and the available knowledge of the venom action and composition of allied taxa, as presented in this annotated checklist, we propose a classification of the Ecuadorian scorpion fauna as follows:

- Species in genera *Hadruroides*, which are mainly coastal (Fig. 4), would produce severe accidents only if envenoming occurs by a central pathway. Local manifestations such as intense pain, edema and ulceration are expected [100]. Venoms of *Hadruroides* spp. contain cytotoxic components that may produce heart muscle disruption. Sting by *Brachistosternus* spp., also a coastal species in Ecuador [with the exception of *B.* (*B.*) *pegnai*, restricted to Carchi], and recognized by their generally clear and yellowish coloration, produces intense pain in humans without further complications, although some venoms contain neurotoxins that can produce autonomic effects [35].
- Species in genera *Centruroides* and *Teuthraustes* inhabiting Ecuador, particularly *C. margaritatus* and *T. atramentarius* (Fig. 3) appear not to produce venoms significantly toxic to humans. For instance, envenoming by the *C. margaritatus* population inhabiting the surroundings of Cali, Colombia – morphologically related to the populations prevalent in the coastal regions of Ecuador [101] – produce intense pain, some discoloration at the sting site, and fever (average 39 °C) 3 to 4 h after the accident, without further complications [52]. *T. atramentarius* appears to be weakly ly toxic to humans [89].
- Species in the genus *Tityus* differ in their venom toxicity towards humans depending on the morphological group to which they pertain:
- Species belonging to the subgenus *Archaeotityus* (encompassing the “*Tityus clathratus*” group) comprise small (18–40 mm), highly pigmented scorpions capable of producing toxins with a fingerprint structurally similar to noxious *Tityus* spp. [e.g. *Tityus* (*Archaeotityus*) *clathratus* Koch] but they are not considered medically significant due to the low amount of venom injected [102]. In Ecuador these species are *T*. (*A*.) *julianae*, *T*. (*A*.) *intermedius*, *T*. (*A*.) *bastosi*, *T*. (*A*.) *pugilator*, and *T*. (*A*.) *silvestris*.
- Species in the subgenus *Atreus* in Ecuador belong either to the morphological groups “*Tityus forcípula*” [*T*. (*A*.) *forcipula*, *T*. (*A*.) *crassicauda*, and *T*. (*A*.) *timendus*], “*Tityus androcottoides*” [*T*. (*A*.) *ythieri*], or “*Tityus asthenes*” [*T*. (*A*.) *asthenes*]. These species seem to be restricted to lowland rain forests of the piedmont east and west of the Andes. Most severe/lethal envenoming cases in northern South America, the Amazonian region, and Central America are due to species in this subgenus [47, 55, 56]. The medically significant Ecuadorian species are most probably all contained within this subgenus.
- Species in the subgenus *Tityus* in Ecuador mainly belong to the “*Tityus bolivianus*” complex [*T*. (*T*.) *demangei*, *T*. (*T*.) *ecuadorensis*, *T*. (*T*.) *roigi*, *T*. (*T*.) *simonsi*, and *T*. (*T*.) *jussarae*], with the exception of *T*. (*T*.) *gasci*. This complex contains species mostly inhabiting the inter-Andean valleys in the “Sierra” region [18]. In contrast to the “*Tityus asthenes*” and “*Tityus forcipula*” complexes, no species within the “*Tityus bolivianus*” group have yet been reported to present medical significance in Ecuador or the countries where this complex is also prevalent (Peru, Bolivia, Argentina).
- Species of the genera *Centruroides* (*C. exsul*) and *Hadruroides* [*H*. (*L*.) *galapagoensis*] inhabiting the Galápagos archipelago are not considered dangerous to humans although their sting is painful [103].

As severe envenoming cases have been recorded from east of the Ecuadorian Andes, a study of the immunological reactivity of venoms from medically significant species towards available scorpion antivenoms is required to establish their neutralization capacity or whether new antibodies are needed [24]. In this sense, only three anti-*Tityus* antivenoms are available: anti-*Tityus* (*Atreus*) *discrepans* (Venezuela), anti-*Tityus* (*Tityus*) *serrulatus* (Brazil), and anti-*Tityus* (*Tityus*) *trivitattus* (Argentina) [21, 55]. More collection work is necessary in regions still poorly sampled, such as the provinces of Pastaza and Zamora Chinchipe where no species have been reported thus far (Fig. 1). In this context, it seems reasonable to assume that a significant part of the Ecuadorian scorpion fauna (and their molecules) is still undiscovered, as pointed out recently in relation to other arachnid groups [104].
